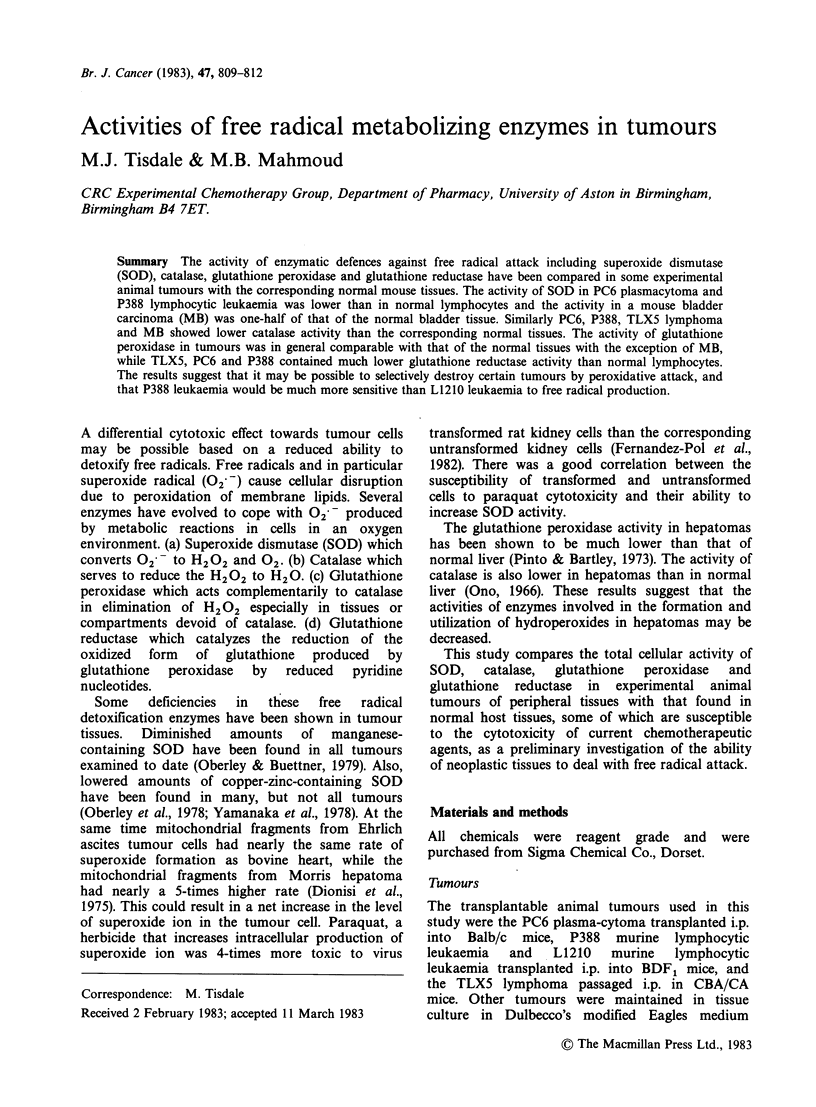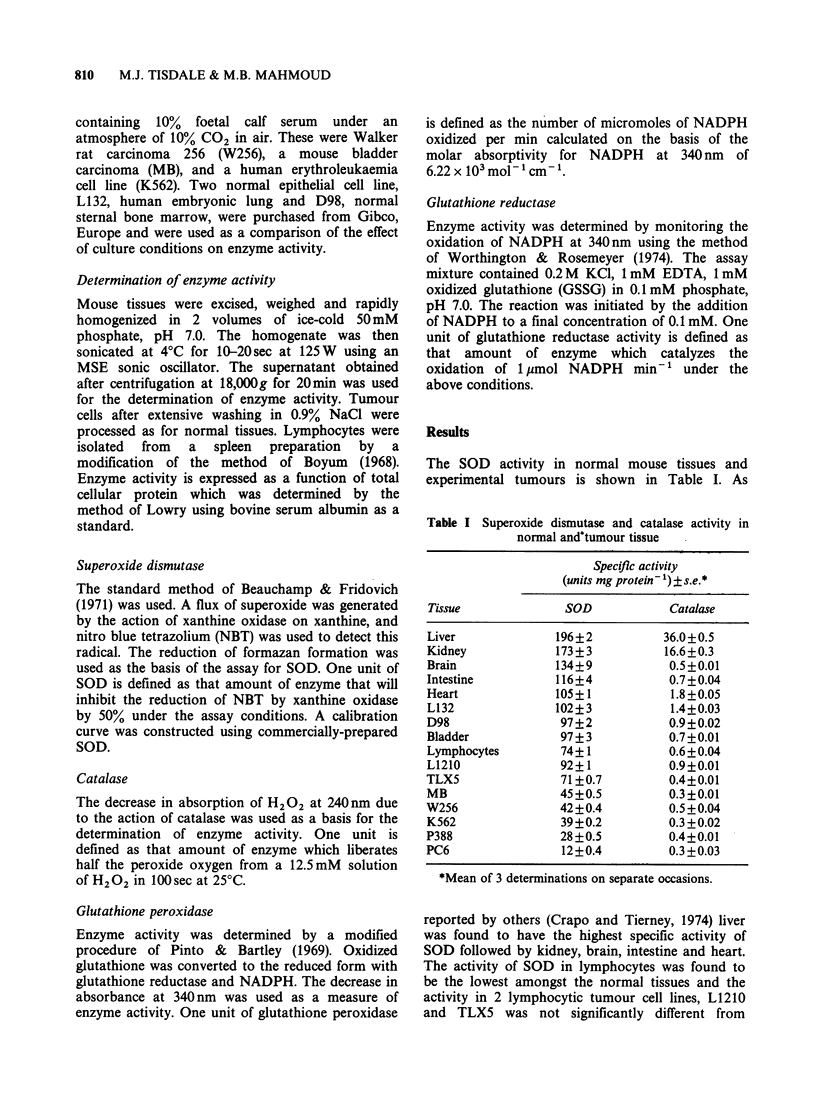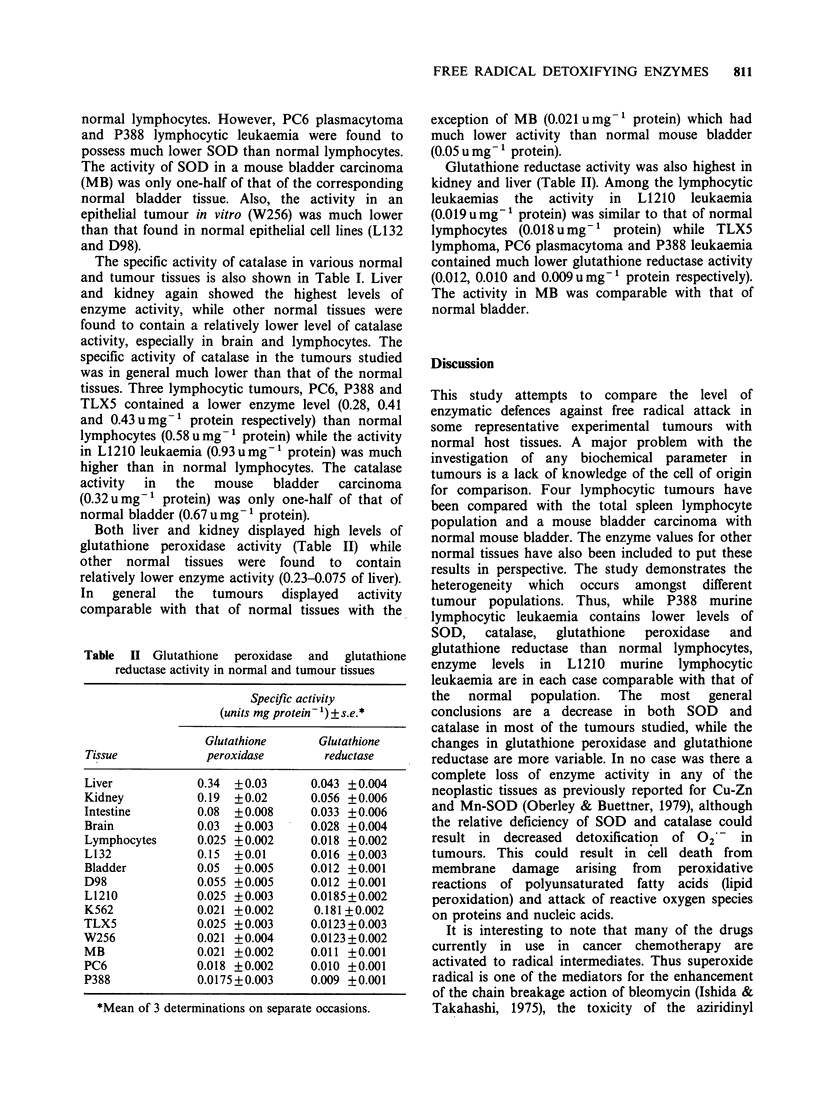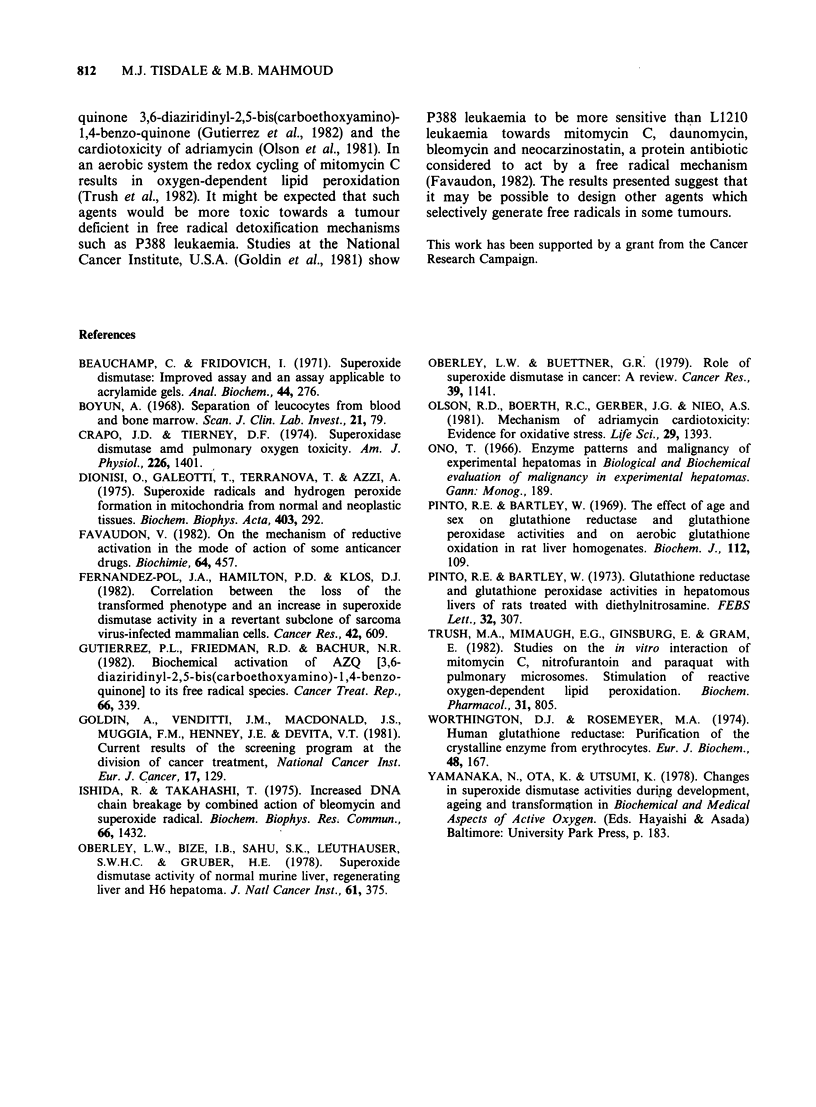# Activities of free radical metabolizing enzymes in tumours.

**DOI:** 10.1038/bjc.1983.135

**Published:** 1983-06

**Authors:** M. J. Tisdale, M. B. Mahmoud

## Abstract

The activity of enzymatic defences against free radical attack including superoxide dismutase (SOD), catalase, glutathione peroxidase and glutathione reductase have been compared in some experimental animal tumours with the corresponding normal mouse tissues. The activity of SOD in PC6 plasmacytoma and P388 lymphocytic leukaemia was lower than in normal lymphocytes and the activity in a mouse bladder carcinoma (MB) was one-half of that of the normal bladder tissue. Similarly PC6, P388, TLX5 lymphoma and MB showed lower catalase activity than the corresponding normal tissues. The activity of glutathione peroxidase in tumours was in general comparable with that of the normal tissues with the exception of MB, while TLX5, PC6 and P388 contained much lower glutathione reductase activity than normal lymphocytes. The results suggest that it may be possible to selectively destroy certain tumours by peroxidative attack, and that P388 leukaemia would be much more sensitive than L1210 leukaemia to free radical production.


*Br. J. Cancer* (1983), **47**, 809–812

### Activities of free radical metabolizing enzymes in tumours

M.J. Tisdale & M.B. Mahmoud

*CRC Experimental Chemotherapy Group, Department of Pharmacy, University of Aston in Birmingham, Birmingham B4 7ET.*

**Summary** The activity of enzymatic defences against free radical attack including superoxide dismutase (SOD), catalase, glutathione peroxidase and glutathione reductase have been compared in some experimental animal tumours with the corresponding normal mouse tissues. The activity of SOD in PC6 plasmacytoma and P388 lymphocytic leukaemia was lower than in normal lymphocytes and the activity in a mouse bladder carcinoma (MB) was one-half of that of the normal bladder tissue. Similarly PC6, P388, TLX5 lymphoma and MB showed lower catalase activity than the corresponding normal tissues. The activity of glutathione peroxidase in tumours was in general comparable with that of the normal tissues with the exception of MB, while TLX5, PC6 and P388 contained much lower glutathione reductase activity than normal lymphocytes. The results suggest that it may be possible to selectively destroy certain tumours by peroxidative attack, and that P388 leukaemia would be much more sensitive than L1210 leukaemia to free radical production.

A differential cytotoxic effect towards tumour cells may be possible based on a reduced ability to detoxify free radicals. Free radicals and in particular superoxide radical ($O_2^{\cdot -}$) cause cellular disruption due to peroxidation of membrane lipids. Several enzymes have evolved to cope with $O_2^{\cdot -}$ produced by metabolic reactions in cells in an oxygen environment. (a) Superoxide dismutase (SOD) which converts $O_2^{\cdot -}$ to $H_2O_2$ and $O_2$. (b) Catalase which serves to reduce the $H_2O_2$ to $H_2O$. (c) Glutathione peroxidase which acts complementarily to catalase in elimination of $H_2O_2$ especially in tissues or compartments devoid of catalase. (d) Glutathione reductase which catalyzes the reduction of the oxidized form of glutathione produced by glutathione peroxidase by reduced pyridine nucleotides.

Some deficiencies in these free radical detoxification enzymes have been shown in tumour tissues. Diminished amounts of manganese-containing SOD have been found in all tumours examined to date (Oberley & Buettner, 1979). Also, lowered amounts of copper-zinc-containing SOD have been found in many, but not all tumours (Oberley *et al.*, 1978; Yamanaka *et al.*, 1978). At the same time mitochondrial fragments from Ehrlich ascites tumour cells had nearly the same rate of superoxide formation as bovine heart, while the mitochondrial fragments from Morris hepatoma had nearly a 5-times higher rate (Dionisi *et al.*, 1975). This could result in a net increase in the level of superoxide ion in the tumour cell. Paraquat, a herbicide that increases intracellular production of superoxide ion was 4-times more toxic to virus transformed rat kidney cells than the corresponding untransformed kidney cells (Fernandez-Pol *et al.*, 1982). There was a good correlation between the susceptibility of transformed and untransformed cells to paraquat cytotoxicity and their ability to increase SOD activity.

The glutathione peroxidase activity in hepatomas has been shown to be much lower than that of normal liver (Pinto & Bartley, 1973). The activity of catalase is also lower in hepatomas than in normal liver (Ono, 1966). These results suggest that the activities of enzymes involved in the formation and utilization of hydroperoxides in hepatomas may be decreased.

This study compares the total cellular activity of SOD, catalase, glutathione peroxidase and glutathione reductase in experimental animal tumours of peripheral tissues with that found in normal host tissues, some of which are susceptible to the cytotoxicity of current chemotherapeutic agents, as a preliminary investigation of the ability of neoplastic tissues to deal with free radical attack.

### Materials and methods

All chemicals were reagent grade and were purchased from Sigma Chemical Co., Dorset.

### *Tumours*

The transplantable animal tumours used in this study were the PC6 plasma-cytoma transplanted i.p. into Balb/c mice, P388 murine lymphocytic leukaemia and L1210 murine lymphocytic leukaemia transplanted i.p. into $BDF_1$ mice, and the TLX5 lymphoma passaged i.p. in CBA/CA mice. Other tumours were maintained in tissue culture in Dulbecco's modified Eagles medium

Correspondence: M. Tisdale

Received 2 February 1983; accepted 11 March 1983

© The Macmillan Press Ltd., 1983

containing 10% foetal calf serum under an atmosphere of 10% $CO_2$ in air. These were Walker rat carcinoma 256 (W256), a mouse bladder carcinoma (MB), and a human erythroleukaemia cell line (K562). Two normal epithelial cell line, L132, human embryonic lung and D98, normal sternal bone marrow, were purchased from Gibco, Europe and were used as a comparison of the effect of culture conditions on enzyme activity.

*Determination of enzyme activity*

Mouse tissues were excised, weighed and rapidly homogenized in 2 volumes of ice-cold 50 mM phosphate, pH 7.0. The homogenate was then sonicated at 4°C for 10–20 sec at 125 W using an MSE sonic oscillator. The supernatant obtained after centrifugation at 18,000 *g* for 20 min was used for the determination of enzyme activity. Tumour cells after extensive washing in 0.9% NaCl were processed as for normal tissues. Lymphocytes were isolated from a spleen preparation by a modification of the method of Boyum (1968). Enzyme activity is expressed as a function of total cellular protein which was determined by the method of Lowry using bovine serum albumin as a standard.

*Superoxide dismutase*

The standard method of Beauchamp & Fridovich (1971) was used. A flux of superoxide was generated by the action of xanthine oxidase on xanthine, and nitro blue tetrazolium (NBT) was used to detect this radical. The reduction of formazan formation was used as the basis of the assay for SOD. One unit of SOD is defined as that amount of enzyme that will inhibit the reduction of NBT by xanthine oxidase by 50% under the assay conditions. A calibration curve was constructed using commercially-prepared SOD.

*Catalase*

The decrease in absorption of $H_2O_2$ at 240 nm due to the action of catalase was used as a basis for the determination of enzyme activity. One unit is defined as that amount of enzyme which liberates half the peroxide oxygen from a 12.5 mM solution of $H_2O_2$ in 100 sec at 25°C.

*Glutathione peroxidase*

Enzyme activity was determined by a modified procedure of Pinto & Bartley (1969). Oxidized glutathione was converted to the reduced form with glutathione reductase and NADPH. The decrease in absorbance at 340 nm was used as a measure of enzyme activity. One unit of glutathione peroxidase is defined as the number of micromoles of NADPH oxidized per min calculated on the basis of the molar absorptivity for NADPH at 340 nm of $6.22 \times 10^3 \text{mol}^{-1} \text{cm}^{-1}$.

*Glutathione reductase*

Enzyme activity was determined by monitoring the oxidation of NADPH at 340 nm using the method of Worthington & Rosemeyer (1974). The assay mixture contained 0.2 M KCl, 1 mM EDTA, 1 mM oxidized glutathione (GSSG) in 0.1 mM phosphate, pH 7.0. The reaction was initiated by the addition of NADPH to a final concentration of 0.1 mM. One unit of glutathione reductase activity is defined as that amount of enzyme which catalyzes the oxidation of 1 $\mu$mol NADPH $\text{min}^{-1}$ under the above conditions.

### Results

The SOD activity in normal mouse tissues and experimental tumours is shown in Table I. As reported by others (Crapo and Tierney, 1974) liver was found to have the highest specific activity of SOD followed by kidney, brain, intestine and heart. The activity of SOD in lymphocytes was found to be the lowest amongst the normal tissues and the activity in 2 lymphocytic tumour cell lines, L1210 and TLX5 was not significantly different from

**Table I** Superoxide dismutase and catalase activity in normal and tumour tissue

| | *Specific activity (units mg protein$^{-1}$)$\pm$s.e.** | |
|---|---|---|
| *Tissue* | *SOD* | *Catalase* |
| Liver | 196±2 | 36.0±0.5 |
| Kidney | 173±3 | 16.6±0.3 |
| Brain | 134±9 | 0.5±0.01 |
| Intestine | 116±4 | 0.7±0.04 |
| Heart | 105±1 | 1.8±0.05 |
| L132 | 102±3 | 1.4±0.03 |
| D98 | 97±2 | 0.9±0.02 |
| Bladder | 97±3 | 0.7±0.01 |
| Lymphocytes | 74±1 | 0.6±0.04 |
| L1210 | 92±1 | 0.9±0.01 |
| TLX5 | 71±0.7 | 0.4±0.01 |
| MB | 45±0.5 | 0.3±0.01 |
| W256 | 42±0.4 | 0.5±0.04 |
| K562 | 39±0.2 | 0.3±0.02 |
| P388 | 28±0.5 | 0.4±0.01 |
| PC6 | 12±0.4 | 0.3±0.03 |

*Mean of 3 determinations on separate occasions.

normal lymphocytes. However, PC6 plasmacytoma and P388 lymphocytic leukaemia were found to possess much lower SOD than normal lymphocytes. The activity of SOD in a mouse bladder carcinoma (MB) was only one-half of that of the corresponding normal bladder tissue. Also, the activity in an epithelial tumour *in vitro* (W256) was much lower than that found in normal epithelial cell lines (L132 and D98).

The specific activity of catalase in various normal and tumour tissues is also shown in Table I. Liver and kidney again showed the highest levels of enzyme activity, while other normal tissues were found to contain a relatively lower level of catalase activity, especially in brain and lymphocytes. The specific activity of catalase in the tumours studied was in general much lower than that of the normal tissues. Three lymphocytic tumours, PC6, P388 and TLX5 contained a lower enzyme level (0.28, 0.41 and 0.43 u $mg^{-1}$ protein respectively) than normal lymphocytes (0.58 u $mg^{-1}$ protein) while the activity in L1210 leukaemia (0.93 u $mg^{-1}$ protein) was much higher than in normal lymphocytes. The catalase activity in the mouse bladder carcinoma (0.32 u $mg^{-1}$ protein) was only one-half of that of normal bladder (0.67 u $mg^{-1}$ protein).

Both liver and kidney displayed high levels of glutathione peroxidase activity (Table II) while other normal tissues were found to contain relatively lower enzyme activity (0.23–0.075 of liver). In general the tumours displayed activity comparable with that of normal tissues with the exception of MB (0.021 u $mg^{-1}$ protein) which had much lower activity than normal mouse bladder (0.05 u $mg^{-1}$ protein).

Glutathione reductase activity was also highest in kidney and liver (Table II). Among the lymphocytic leukaemias the activity in L1210 leukaemia (0.019 u $mg^{-1}$ protein) was similar to that of normal lymphocytes (0.018 u $mg^{-1}$ protein) while TLX5 lymphoma, PC6 plasmacytoma and P388 leukaemia contained much lower glutathione reductase activity (0.012, 0.010 and 0.009 u $mg^{-1}$ protein respectively). The activity in MB was comparable with that of normal bladder.

**Table II** Glutathione peroxidase and glutathione reductase activity in normal and tumour tissues

| Tissue | *Specific activity (units mg protein$^{-1}$)$\pm$s.e.** Glutathione peroxidase | Glutathione reductase |
|---|---|---|
| Liver | 0.34 ±0.03 | 0.043 ±0.004 |
| Kidney | 0.19 ±0.02 | 0.056 ±0.006 |
| Intestine | 0.08 ±0.008 | 0.033 ±0.006 |
| Brain | 0.03 ±0.003 | 0.028 ±0.004 |
| Lymphocytes | 0.025 ±0.002 | 0.018 ±0.002 |
| L132 | 0.15 ±0.01 | 0.016 ±0.003 |
| Bladder | 0.05 ±0.005 | 0.012 ±0.001 |
| D98 | 0.055 ±0.005 | 0.012 ±0.001 |
| L1210 | 0.025 ±0.003 | 0.0185±0.002 |
| K562 | 0.021 ±0.002 | 0.181±0.002 |
| TLX5 | 0.025 ±0.003 | 0.0123±0.003 |
| W256 | 0.021 ±0.004 | 0.0123±0.002 |
| MB | 0.021 ±0.002 | 0.011 ±0.001 |
| PC6 | 0.018 ±0.002 | 0.010 ±0.001 |
| P388 | 0.0175±0.003 | 0.009 ±0.001 |

*Mean of 3 determinations on separate occasions.

### Discussion

This study attempts to compare the level of enzymatic defences against free radical attack in some representative experimental tumours with normal host tissues. A major problem with the investigation of any biochemical parameter in tumours is a lack of knowledge of the cell of origin for comparison. Four lymphocytic tumours have been compared with the total spleen lymphocyte population and a mouse bladder carcinoma with normal mouse bladder. The enzyme values for other normal tissues have also been included to put these results in perspective. The study demonstrates the heterogeneity which occurs amongst different tumour populations. Thus, while P388 murine lymphocytic leukaemia contains lower levels of SOD, catalase, glutathione peroxidase and glutathione reductase than normal lymphocytes, enzyme levels in L1210 murine lymphocytic leukaemia are in each case comparable with that of the normal population. The most general conclusions are a decrease in both SOD and catalase in most of the tumours studied, while the changes in glutathione peroxidase and glutathione reductase are more variable. In no case was there a complete loss of enzyme activity in any of the neoplastic tissues as previously reported for Cu-Zn and Mn-SOD (Oberley & Buettner, 1979), although the relative deficiency of SOD and catalase could result in decreased detoxification of $O_2^{\cdot -}$ in tumours. This could result in cell death from membrane damage arising from peroxidative reactions of polyunsaturated fatty acids (lipid peroxidation) and attack of reactive oxygen species on proteins and nucleic acids.

It is interesting to note that many of the drugs currently in use in cancer chemotherapy are activated to radical intermediates. Thus superoxide radical is one of the mediators for the enhancement of the chain breakage action of bleomycin (Ishida & Takahashi, 1975), the toxicity of the aziridinyl quinone 3,6-diaziridinyl-2,5-bis(carboethoxyamino)-1,4-benzo-quinone (Gutierrez *et al.*, 1982) and the cardiotoxicity of adriamycin (Olson *et al.*, 1981). In an aerobic system the redox cycling of mitomycin C results in oxygen-dependent lipid peroxidation (Trush *et al.*, 1982). It might be expected that such agents would be more toxic towards a tumour deficient in free radical detoxification mechanisms such as P388 leukaemia. Studies at the National Cancer Institute, U.S.A. (Goldin *et al.*, 1981) show P388 leukaemia to be more sensitive than L1210 leukaemia towards mitomycin C, daunomycin, bleomycin and neocarzinostatin, a protein antibiotic considered to act by a free radical mechanism (Favaudon, 1982). The results presented suggest that it may be possible to design other agents which selectively generate free radicals in some tumours.

This work has been supported by a grant from the Cancer Research Campaign.

### References

BEAUCHAMP, C. & FRIDOVICH, I. (1971). Superoxide dismutase: Improved assay and an assay applicable to acrylamide gels. *Anal. Biochem.*, **44**, 276.

BOYUN, A. (1968). Separation of leucocytes from blood and bone marrow. *Scan. J. Clin. Lab. Invest.*, **21**, 79.

CRAPO, J.D. & TIERNEY, D.F. (1974). Superoxidase dismutase amd pulmonary oxygen toxicity. *Am. J. Physiol.*, **226**, 1401.

DIONISI, O., GALEOTTI, T., TERRANOVA, T. & AZZI, A. (1975). Superoxide radicals and hydrogen peroxide formation in mitochondria from normal and neoplastic tissues. *Biochem. Biophys. Acta*, **403**, 292.

FAVAUDON, V. (1982). On the mechanism of reductive activation in the mode of action of some anticancer drugs. *Biochimie*, **64**, 457.

FERNANDEZ-POL, J.A., HAMILTON, P.D. & KLOS, D.J. (1982). Correlation between the loss of the transformed phenotype and an increase in superoxide dismutase activity in a revertant subclone of sarcoma virus-infected mammalian cells. *Cancer Res.*, **42**, 609.

GUTIERREZ, P.L., FRIEDMAN, R.D. & BACHUR, N.R. (1982). Biochemical activation of AZQ [3,6-diaziridinyl-2,5-bis(carboethoxyamino)-1,4-benzo-quinone] to its free radical species. *Cancer Treat. Rep.*, **66**, 339.

GOLDIN, A., VENDITTI, J.M., MACDONALD, J.S., MUGGIA, F.M., HENNEY, J.E. & DEVITA, V.T. (1981). Current results of the screening program at the division of cancer treatment, *National Cancer Inst. Eur. J. Cancer*, **17**, 129.

ISHIDA, R. & TAKAHASHI, T. (1975). Increased DNA chain breakage by combined action of bleomycin and superoxide radical. *Biochem. Biophys. Res. Commun.*, **66**, 1432.

OBERLEY, L.W., BIZE, I.B., SAHU, S.K., LEUTHAUSER, S.W.H.C. & GRUBER, H.E. (1978). Superoxide dismutase activity of normal murine liver, regenerating liver and H6 hepatoma. *J. Natl Cancer Inst.*, **61**, 375.

OBERLEY, L.W. & BUETTNER, G.R. (1979). Role of superoxide dismutase in cancer: A review. *Cancer Res.*, **39**, 1141.

OLSON, R.D., BOERTH, R.C., GERBER, J.G. & NIEO, A.S. (1981). Mechanism of adriamycin cardiotoxicity: Evidence for oxidative stress. *Life Sci.*, **29**, 1393.

ONO, T. (1966). Enzyme patterns and malignancy of experimental hepatomas in *Biological and Biochemical evaluation of malignancy in experimental hepatomas. Gann: Monog.*, 189.

PINTO, R.E. & BARTLEY, W. (1969). The effect of age and sex on glutathione reductase and glutathione peroxidase activities and on aerobic glutathione oxidation in rat liver homogenates. *Biochem. J.*, **112**, 109.

PINTO, R.E. & BARTLEY, W. (1973). Glutathione reductase and glutathione peroxidase activities in hepatomous livers of rats treated with diethylnitrosamine. *FEBS Lett.*, **32**, 307.

TRUSH, M.A., MIMAUGH, E.G., GINSBURG, E. & GRAM, E. (1982). Studies on the *in vitro* interaction of mitomycin C, nitrofurantoin and paraquat with pulmonary microsomes. Stimulation of reactive oxygen-dependent lipid peroxidation. *Biochem. Pharmacol.*, **31**, 805.

WORTHINGTON, D.J. & ROSEMEYER, M.A. (1974). Human glutathione reductase: Purification of the crystalline enzyme from erythrocytes. *Eur. J. Biochem.*, **48**, 167.

YAMANAKA, N., OTA, K. & UTSUMI, K. (1978). Changes in superoxide dismutase activities during development, ageing and transformation in *Biochemical and Medical Aspects of Active Oxygen.* (Eds. Hayaishi & Asada) Baltimore: University Park Press, p. 183.